# Optimization Studies on Recovery of Metals from Printed Circuit Board Waste

**DOI:** 10.1155/2018/1067512

**Published:** 2018-11-01

**Authors:** P. Sivakumar, D. Prabhakaran, M. Thirumarimurugan

**Affiliations:** Department of Chemical Engineering, Coimbatore Institute of Technology, Coimbatore 641 014, Tamil Nadu, India

## Abstract

The aim of the study was to recover copper and lead metal from waste printed circuit boards (PCBs). The electrowinning method is found to be an effective recycling process to recover copper and lead metal from printed circuit board wastes. In order to simplify the process with affordable equipment, a simple ammonical leaching operation method was adopted. The selected PCBs were incinerated into fine ash powder at 500°C for 1 hour in the pyrolysis reactor. Then, the fine ash powder was subjected to acid-leaching process to recover the metals with varying conditions like acid-base concentration, electrode combination, and leaching time. The relative electrolysis solution of 0.1 M lead nitrate for lead and 0.1 M copper sulphate for copper was used to extract metals from PCBs at room temperature. The amount of lead and copper extracted from the process was determined by an atomic absorption spectrophotometer, and results found were 73.29% and 82.17%, respectively. Further, the optimum conditions for the recovery of metals were determined by using RSM software. The results showed that the percentage of lead and copper recovery were 78.25% and 89.1% should be 4 hrs 10 A/dm^2^.

## 1. Introduction

Recycling of e-waste is an important subject not only from the point of waste treatment but also from the recovery aspect of valuable materials [1–4]. Among the resources in e-waste, metals contribute more than 95% of the materials market value. Hence, the recovery of valuable metals is the inherent motive in e-waste disposal. In the past decades, many techniques for recovering valuable metals from e-waste have been developed such as gravity separation, magnetic separation, and electrostatic separation [5] synthesis of CuCl with e-waste, separation of PCBs with organic solvent method [6, 7], cyanide and noncyanide lixiviants leaching methods, ammonium persulfate leaching bioleaching methods [8–10], or a combination of these approaches. Among those methods, hydrometallurgical methods are more accurate, predictable, and controllable [11]. Therefore, hydrometallurgical techniques are most active in the research of valuable metal recovery from electronic scraps in the past two decades. However, traditional hydrometallurgical methods are acid dependent, time-consuming, and inefficient for simultaneous recovery of precious metals. Remarkably, a large amount of corrosive or toxic reagents, such as aqua regia, nitric acid, cyanide and halide, are consumed, producing large quantities of toxic and corrosive fumes or solution [12, 13]. Therefore, it is necessary to seek a more environmental friendly method for the recovery of valuable metals from e-wastes. Hydrometallurgical methods are used in the upgrading and refining stages of the recycling chain [14–16]. In this research article, the recovery of lead and copper metals from e-waste is widely investigated. The PCBs were converted into fine ash powder and subjected to electrowinning process for the recovery of metals. The experimental results were determined by EDS and AAS, respectively. Furthermore, the experimental results are validated through RSM software at different parameters like acid-base concentration, electrode combination, and leaching time [17–22].

## 2. Materials and Methods

### 2.1. Materials

The computer PCBs were collected from various sources for the recovery of metals. The collected PCBs were crushed using roll crusher and powdered by a hammer mill. The crushed PCBs were incarnated through pyrolysis to avoid side reaction in the leaching process with the electrolyte solution. The optimum condition of the pyrolysis reactor was 500°C in atmospheric pressure for 1 h where the epoxy resins and polymers were volatized at the temperature less than 500°C. The volatized contents were condensed and collected separately. The ferrous materials present in the obtained ash were separated by a magnetic separator.

### 2.2. Electrowinning Process

**Figure 1:**
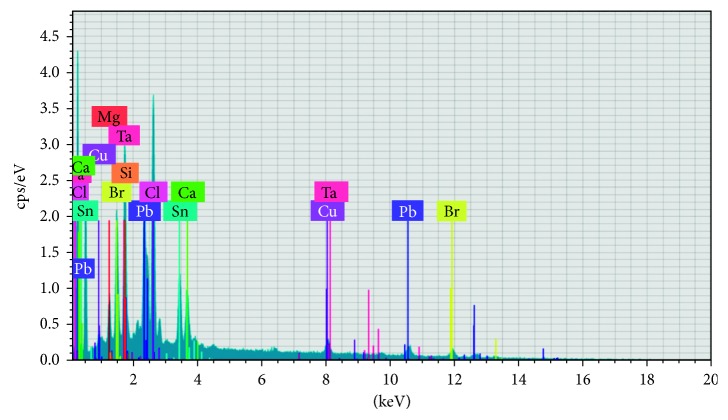
Initial analysis of raw materials.

**Figure 2:**
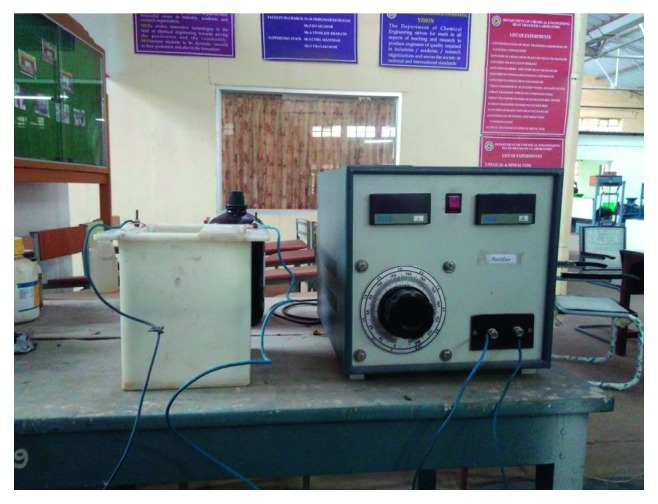
Experimental setup of electrowinning process.

The fine ash powder was treated with aqua regia solution (3 : 1 ratio of HCl and HNO_3_) in the incineration chamber in order to avoid the liberation of toxic fumes. Then the precipitated salts obtained from the leaching was analyzed by EDS to determine the composition of metal present in the salts (Figure 1). The electrowinning setup consists of bath arrangement and amplifier. The bath having two slots for the anode and cathode fixing and the electrode is connected with amplifier, and the current density was varied through the amplifier (Figure 2).

### 2.3. Extraction Process of Lead

About 25 g of incinerated fine ash was added into the acid bath followed by the addition of ammonical electrolyte solution. The current density was set to 1 to 10 (A/dm^2^). The solution was agitated at regular interval to get an effective electrodeposition:

(1) $$\begin{array}{c} \text{Pb}+4\text{HCl}⟶{\text{PbCl}}_{4}+{2\text{H}}_{2} \end{array}$$

After the stipulated time of operation, pure lead was deposited on lead cathode. The deposited elements were scrapped and stored in an air tight container. The recovered lead quantitated from the EDS method. The spent acid left with mud filtered at pH 6–10 was stored in a glass container for further treatment.

### 2.4. Extraction Process of Copper

**Figure 3:**
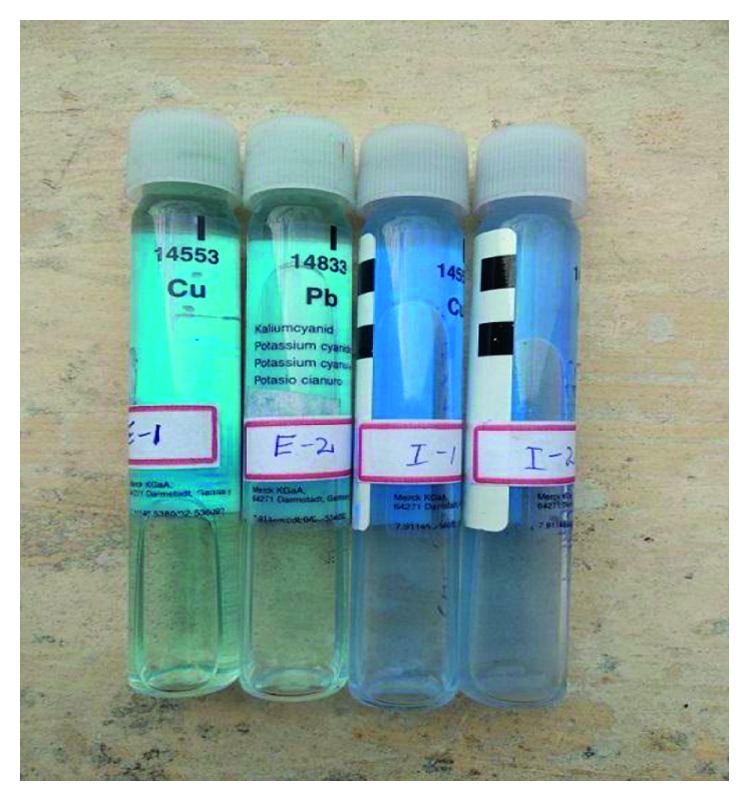
Bath solutions of copper and lead.

About 25 g of incinerated fine ash was added into the acid bath followed by the addition of ammonical electrolyte solution. The current density was set to 1 to 10 (A/dm^2^). The solution was agitated at regular intervals to get an effective electrodeposition. After the stipulated time of operation, pure copper (cupric) was deposited on the cathode and impure copper (cuprous ion) were deposited on the anode. The deposited elements were scrapped and stored in an air tight container. The recovered copper quantitated from the EDS method. The spent acid left with mud (nonleached elements) was filtered (pH–8.4) and were stored in a glass container for further treatment (Figure 3):

(2) $$\begin{array}{c} 2{\text{Cu}}^{2+}\left(\text{aq}\right)+2{\text{H}}_{2}\text{O}\left(\text{l}\right)⟶2\text{Cu}\left(\text{s}\right)+4{\text{H}}^{+}\left(\text{aq}\right)+{\text{O}}_{2}\left(\text{g}\right) \end{array}$$

**Figure 4:**
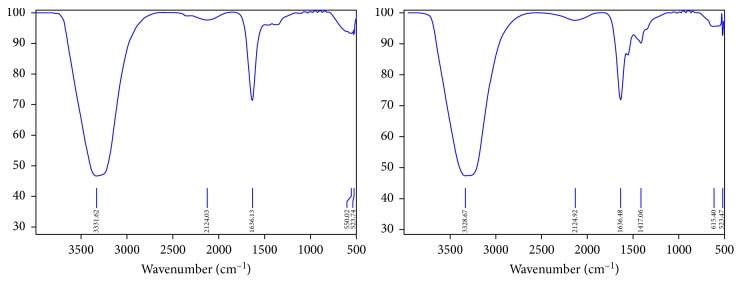
FTIR analysis of bath solution.

The spent solution collected from the electrodeposition was neutralized to 6.9 for the safe disposal as per the standard. Moreover, the presence of any metal in the spent solution was analyzed by Fourier-transform infrared spectroscopy. The results (Figure 4) show that the metallic traces were found to be absent which confirms that all the metals recovered from the ashes deposited on the electrode.

## 3. Results and Discussion

### 3.1. RSM for Lead

**Figure 5:**
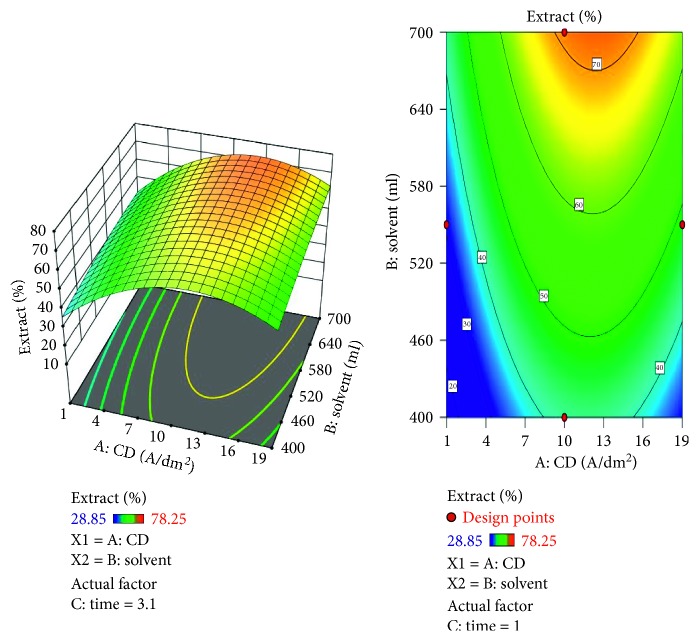
Contour plot for recovery of Lead.

The response surface methodology (RSM) is a statistical modeling technique employed for multiple regression analysis using quantitative data obtained from designed experiments to solve multivariable equations (Table 1). The response surfaces can be visualized as three-dimensional plots that exhibit the response as a function of two factors while keeping the other factors constant. In this above plot, the red zone corresponds to the extract percentage above 85%, yellow zone shows 60 to 70%, and the blue zone confirms below 40% extraction of lead (Figures 5 and 5). The regression equation for the RSM data plots for the lead is

(3) $$\begin{array}{c} \text{Extract}=66.36+9.0175*\text{A}+7.37375*\text{B}+6.42375*\text{C}+1.235*\text{AB}+0.17*\text{AC}+\left(-7.1525*\text{BC}\right)+\left(-18.6187*{\text{A}}^{2}\right)+\left(-1.67125*{\text{B}}^{2}\right)+\left(-1.81625*{\text{C}}^{2}\right). \end{array}$$

The model as a function of coded factor could be utilized to predict the response of each parameter within the given limit. Here, the maximum limit of process parameters (factors) is termed (coded) as +1 and minimum limit is terms (coded) as −1. The modifed equation or coded equation is very much useful in order to find the comparative effect of the process parameters by relating the coefficient of factors. The final equation in terms of actual factors is

(4) $$\begin{array}{c} \text{Extract}=-70.2665+5.06454*\text{CD}+0.201188*\text{solvent}+25.6766*\text{time}+0.000914815*\text{CD}*\text{solvent}+0.0125926*\text{CD}*\text{time}+\left(-0.0317889*\text{solvent}*\text{time}\right)+\left(-0.229861*{\text{CD}}^{2}\right)+\left(-7.42778e-05*{\text{solvent}}^{2}\right)+\left(-0.807222*{\text{time}}^{2}\right). \end{array}$$

Equation (4) in terms of process parameters could be utilized to predict the response for the provided levels of each parameter (Table 2). In this equation, the original units of each parameters should be considered for each levels. In order to evaluate the comparative effect of each factor, the above equation should not be considered since the coefficients are balanced to embrace the units of each parameters. Also, the intercept does not fall at design space center.

### 3.2. Analysis of Variance (ANOVA)

Analysis of variance is used to determine the significant effects of process variables on current efficiency (Table 3) along with the factor coding. The sum of squares is found to be Type III—partial derived from the ANOVA quadratic model. The model *F* value of 4.43 implies the model is significant. A minimum value of 3.12% is possible for the *F* value due to noise. *p* values less than 0.0500 indicate model terms are significant. In this case A, A^2^ are significant model terms. Values greater than 0.1000 indicate the model terms are not significant. If there are many insignificant model terms (not counting those required to support hierarchy), model reduction may improve the model. The lack of fit *F* value of 63.27 implies the lack of fit is significant. There is only a 0.08% chance that a lack of fit *F* value could be large that could occur due to noise. The coefficient represents the expected change in response per unit change in the factor value, when all remaining factors were constant. The intercept in an orthogonal design is the overall average response of all the runs. The coefficients are adjustments around the average factor settings. When the factors are orthogonal, the variance inflation factors (VIFs) are 1; VIFs greater than 1 indicate multicolinearity; the higher the VIF, the more severe the correlation of factors. As a rough rule, VIFs less than 10 are tolerable. Hence, from the data obtained (Table 4), the VIF values of lead are found to be tolerable.

### 3.3. Model Terms

For a standard deviation of 1, the power calculations are performed using response type “continuous,” and parameters are Δ = 2 and *σ* = 1. The power is evaluated over −1 to +1 coded factor space. From (Table 5), the standard errors should be similar to each other in a balanced design. The ideal VIF value should be 1, VIFs above 10 are cause for concern, and VIFs above 100 are cause for alarm, indicating coefficients are poorly estimated due to multicolinearity, where ideal *R*i^2^ is 0.0. High *R*i^2^ means terms are correlated with each other, possibly leading to poor models. If the design has multilinear constraints, then multicolinearity will exist to a greater degree. This inflates the VIFs and the *R*i^2^, rendering these statistics would not perform well. Hence, FDS could be used. Power is an inappropriate tool to evaluate response surface designs. Use prediction-based metrics provided in this program via fraction of design space (FDS) statistics.

### 3.4. Fit Statistics

**Figure 6:**
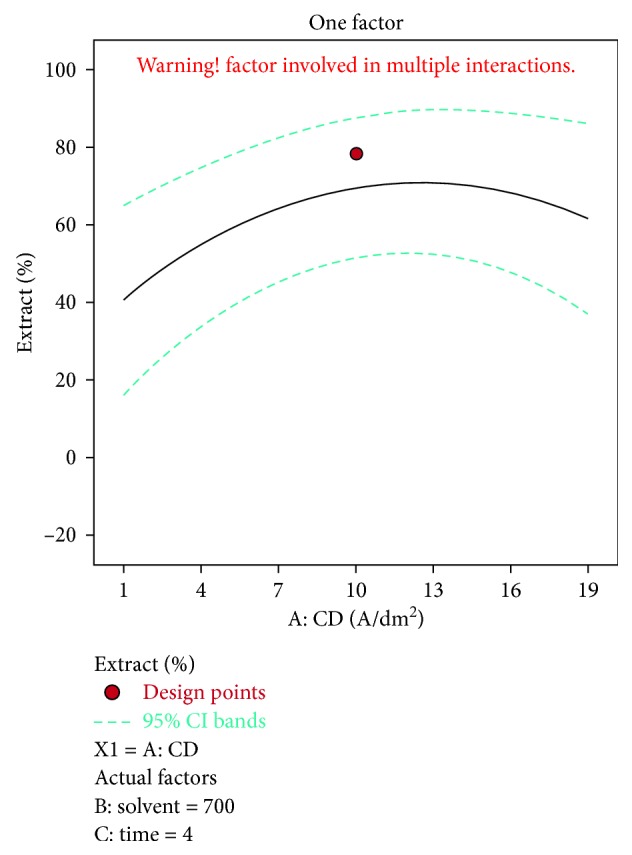
Current density vs extract % for lead.

**Figure 7:**
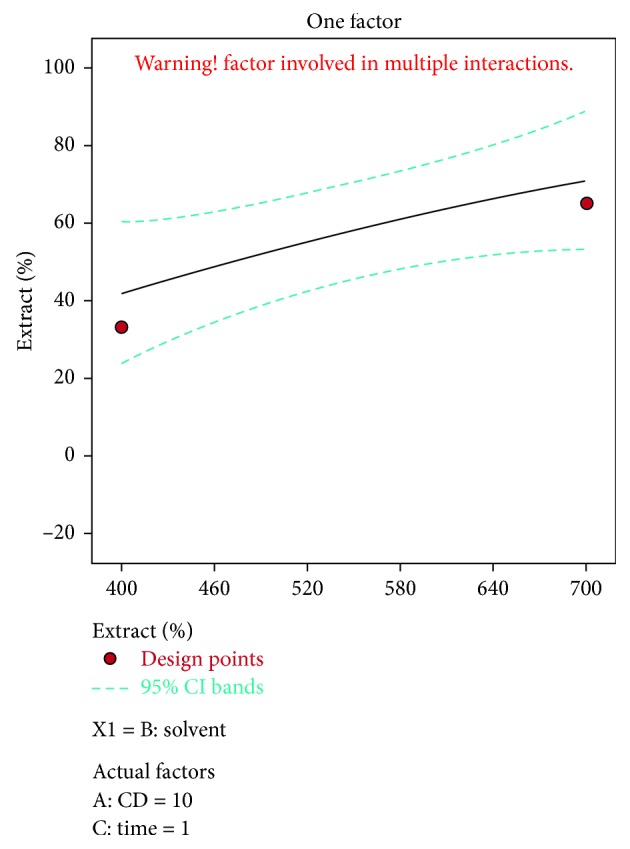
Solvent vs extract % for lead.

**Figure 8:**
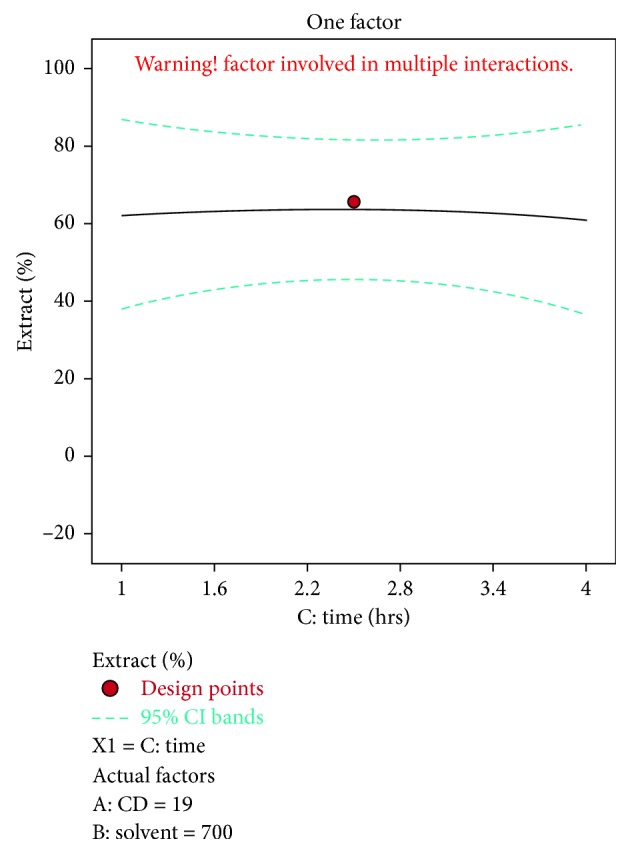
Time vs extract % for lead.

A negative predicted *R*^2^ implies that the overall mean may be a better predictor of the response than the current model. In some cases, a higher order model may also predict better. Adeq. precision measures the signal to noise ratio. A ratio greater than 4 is desirable. The ratio of 5.915 indicates an adequate signal. This model can be used to navigate the design space. The optimization of current efficiency is shown in Figure 6. From the results, it is observed that 69% of lead extract is obtained at current density = 10 A dm^−2^, solvent ratio = 5 : 2, and the electrolysis time = 4 hours (Figures 7 and 8). The significance of regression coefficients were analyzed using the *p*-test and *t*-test. The *p* values are used to check the effect of interaction among the variables. A larger magnitude of *t*-value and a smaller magnitude of *p* value are significant in the corresponding coefficient term. The coefficient of current efficiency and the corresponding *t* and *p* values are shown in Table 6. Finally, the coefficients in the interaction terms for current density-electrolysis time is significant compared to current density-solvent ratio, and current density-electrolysis time.

### 3.5. RSM for Copper

The regression equation for the RSM data plots for the copper is in terms of coded factors form as follows:

(5) $$\begin{array}{c} \text{Extract}\left(\text{E}\right)=48+24*\text{A}+8.25*\text{B}+15.5*\text{C}+0.5*\text{AB}+7.5*\text{AC}+2.5*\text{BC}+\left(-6.5*{\text{A}}^{2}\right)+8*{\text{B}}^{2}+1.5*{\text{C}}^{2}. \end{array}$$

The model (Equation 5) as a function of coded factor could be utilized to predict the response of each parameter within the given limit. Here, the maximum limit of process parameters (factors) is termed(coded) as +1 and minimum limit is termed (coded) as −1. The modified equation or coded equation is very much useful in order to find the comparative effect of the process parameters by relating the coefficient of factors (Table 7).

The final equation in terms of actual factors is

(6) $$\begin{array}{c} \text{Extract}=191.503+2.32716*\text{CD}+\left(-0.773056*\text{solvent}\right)+\left(-11.3333*\text{time}\right)+0.000555556*\text{CD}*\text{solvent}+0.833333*\text{CD}*\text{time}+0.025*\text{solvent}*\text{time}+\left(-0.0802469*{\text{CD}}^{2}\right)+0.0008*{\text{solvent}}^{2}+1.5*{\text{time}}^{2}. \end{array}$$

**Figure 9:**
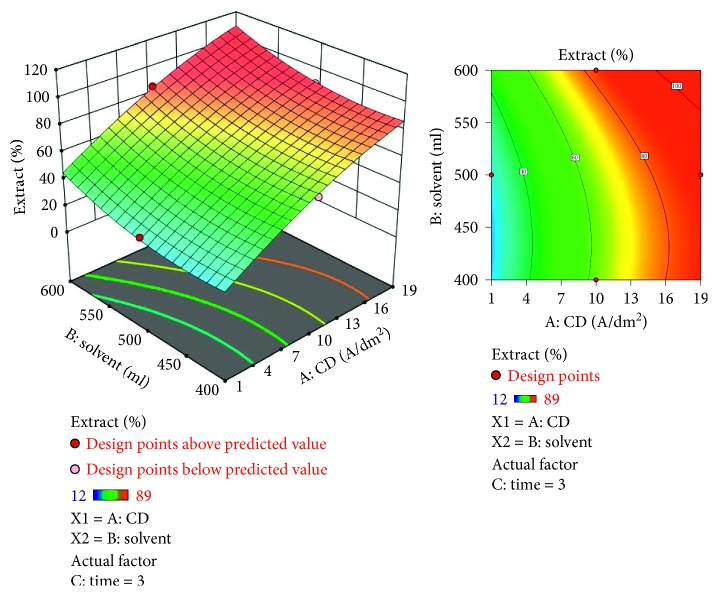
Contour plot for recovery of copper.

Equation (5) in terms of process parameters could be utilized to predict the response for the provided levels of each parameter. In this equation, the original units of each parameters should be considered for each levels. In order to evaluate the comparative effect of each factor, the above equation should not be considered since the coefficients are balanced to embrace the units of each parameters. Also, the intercept does not falls at design space center (Table 8). In this contour plot, the red zone indicates extract percentages above 85%. And yellow and blue zones indicate 60 to 70% and below 40% extraction of copper (Figures 9 and 9).

### 3.6. Analysis of Variance (ANOVA)

Analysis of variance is used to determine the significant effects of process variables on current efficiency along with the factor coding. The sum of squares is found to be Type III—partial derived from the ANOVA quadratic model. The model *F* value of 155.08 in the Table 9 implies the model is significant. A minimum value of 0.01% is possible for the *F* value due to noise. *P* values less than 0.0500 indicate model terms are significant. In this case, A, B, C, AC, A^2^, and B^2^ are significant model terms. Values greater than 0.1000 indicate the model terms are not significant. If there are many insignificant model terms (not counting those required to support hierarchy), model reduction may improve the model. The lack of fit *F* value is nil that implies the lack of fit is significant. The coefficient represents the expected change in response per unit change in factor value, when all remaining factors were constant. The intercept in an orthogonal design is the overall average response of all the runs. The coefficients are adjustments around the average factor settings. When the factors are orthogonal, the VIFs are 1; VIFs greater than 1 indicate multicolinearity; the higher the VIF, the more severe the correlation of factors. As a rough rule, VIFs less than 10 are tolerable. Hence, from the data obtained (Table 10), the VIF Values of lead are found to be tolerable.

### 3.7. Model Terms

For a standard deviation of 1 the power calculations are performed using response type “continuous,” and the parameters are Δ = 2 and *σ* = 1. The power is evaluated over −1 to +1 coded factor space (Table 11). The standard errors should be similar to each other in a balanced design. The ideal VIF value should be 1, VIFs above 10 are cause for concern and VIFs above 100 are cause for alarm, indicating coefficients are poorly estimated due to multicolinearity, where ideal *R*i^2^ is 0.0. High *R*i^2^ means terms are correlated with each other, possibly leading to poor models. If the design has multilinear constraints, then multicolinearity will exist to a greater degree. This inflates the VIFs and the *R*i^2^, rendering these statistics would not perform well. Hence, FDS could be used. Power is an inappropriate tool to evaluate response surface designs. Use prediction-based metrics provided in this program via fraction of design space (FDS) statistics.

### 3.8. Fit Statistics

**Figure 10:**
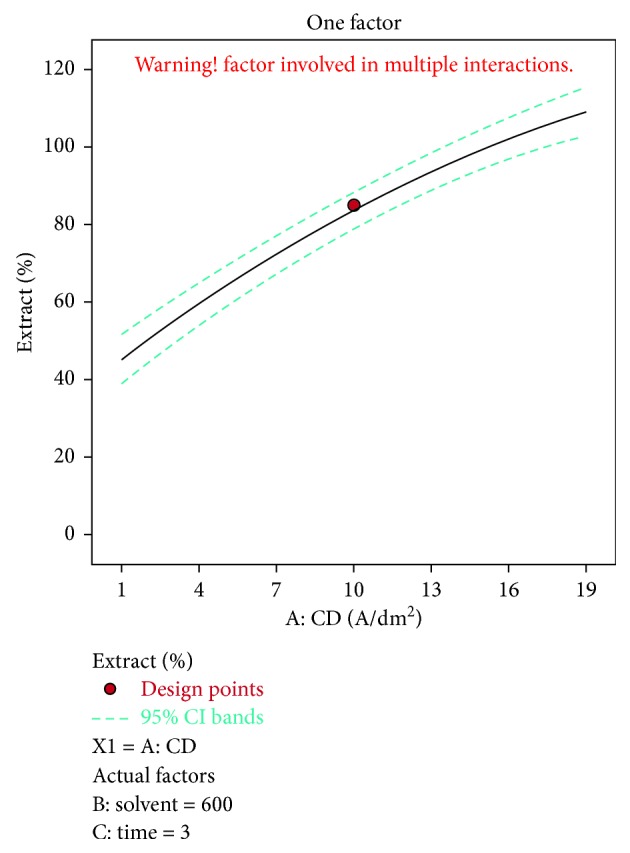
Current density vs extract % for copper.

**Figure 11:**
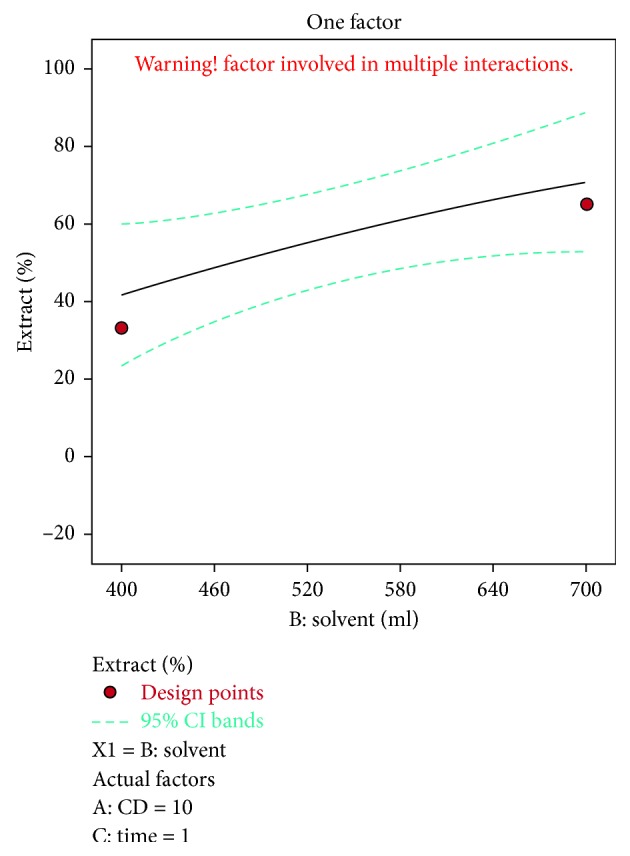
Solvent vs extract % for lead.

**Figure 12:**
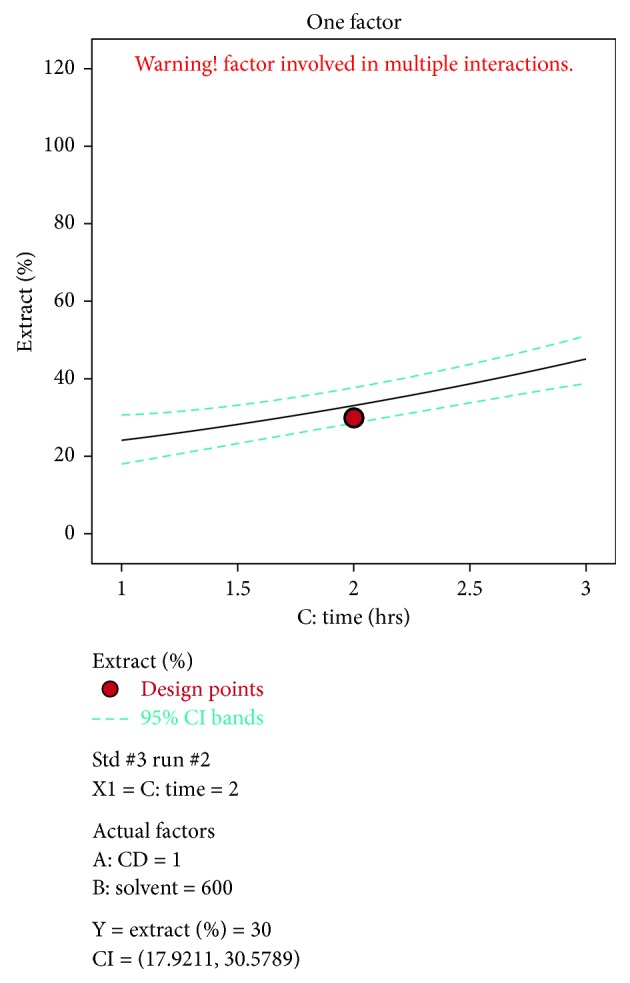
Time vs extract % for lead.

A predicted *R*^2^ implies that the overall mean may be a better predictor of the response than the current model. In some cases, a higher order model may also predict better. Adeq. precision measures the signal to noise ratio. A ratio greater than 4 is desirable. A ratio of 44.9 indicates an adequate signal. This model can be used to navigate the design space. The optimization of current efficiency is shown in Figure 10. The optimum extraction of 69% Cu is obtained at current density = 19 A dm^−2^, solvent ratio = 5 : 2, and electrolysis time = 4 hour (Figures 11 and 12). The significance of regression coefficients was analyzed using the *p*-test and *t*-test. The *p* values are used to check the effect of interaction among the variables. A larger magnitude of *t*-value and a smaller magnitude of *p* value are significant in the corresponding coefficient term. The coefficient of current efficiency and the corresponding *t* and *p* values are shown in (Table 12). Finally, the coefficients in the interaction terms for current density-electrolysis time is significant compared to current density-solvent ratio and current density-electrolysis time.

## 4. Conclusion

The ammonia-lead nitrate and ammonia-copper sulphate system have been employed as a leaching agent for recovery of lead and copper from scraped printed circuit board wastes. A two-stage leaching was employed, wherein the first stage consisted of leaching the scrap board with 0.1 M Pb(NO_3_)_2_ and 0.1 M CuSO_4_ which results in the selective dissolution of lead and copper leaching rate, and other metals was found in lower amounts, respectively. The undissolved residue portion from the leaching stage containing nickel, tin, and silica were leached out in respective treatments. The current efficiency was found to increase with current density and concentration ratio with the contact time in acid bath. Hence, 73.29% lead and 82.17% copper have been successfully recovered from the electrolysis process. And, also by RSM Software prediction, the recovery of lead and copper are as 78.25% and 89.1%, respectively. In addition to the quadratic model equation, ANOVA, model terms, and fit statistics were also tested for the experimental conditions.

## Figures and Tables

**Table 1:** RSM parameters for lead extraction.

Std	Run	Factor 1	Factor 2	Factor 3
		A: CD	B: solvent	C: time
		A/dm^2^	ml	Hrs
1	8	1	400	2.5
2	9	19	400	2.5
3	17	1	700	2.5
4	12	19	700	2.5
5	1	1	550	1
6	14	19	550	1
7	6	1	550	4
8	5	19	550	4
9	4	10	400	1
10	10	10	700	1
11	7	10	400	4
12	11	10	700	4
13	2	10	550	2.5
14	16	10	550	2.5
15	13	10	550	2.5
16	15	10	550	2.5
17	3	10	550	2.5

**Table 2:** Box–Behnken experimental design table for recovery of lead.

Std	Run	Factor 1	Factor 2	Factor 3
		A: CD	B: solvent	C: time
		A/dm^2^	ml	Hrs
1	8	1	400	2.5
2	9	19	400	2.5
3	17	1	700	2.5
4	12	19	700	2.5
5	1	1	550	1
6	14	19	550	1
7	6	1	550	4
8	5	19	550	4
9	4	10	400	1
10	10	10	700	1
11	7	10	400	4
12	11	10	700	4
13	2	10	550	2.5
14	16	10	550	2.5
15	13	10	550	2.5
16	15	10	550	2.5
17	3	10	550	2.5

**Table 3:** ANOVA quadratic model for lead.

Source	Sum of squares	DOF	Mean square	*F* value	*p* value	
*Model*	3152.45	9	350.27	4.43	0.0312	Significant
A-CD	650.52	1	650.52	8.23	0.0240	
B-solvent	434.98	1	434.98	5.51	0.0514	
C-time	330.12	1	330.12	4.18	0.0802	
AB	6.10	1	6.10	0.0772	0.7891	
AC	0.1156	1	0.1156	0.0015	0.9706	
BC	204.63	1	204.63	2.59	0.1516	
A^2^	1459.61	1	1459.61	18.47	0.0036	
B^2^	11.76	1	11.76	0.1489	0.7111	
C^2^	13.89	1	13.89	0.1758	0.6876	
*Residual*	553.05	7	79.01			
Lack of fit	541.64	3	180.55	63.27	0.0008	Significant
Pure error	11.41	4	2.85			
*Total*	3705.50	16				

**Table 4:** Coefficients in terms of coded factors for lead.

Factor	Coefficient estimate	DOF	Standard error	95% CI low	95% CI high	VIF
Intercept	66.36	1	3.98	56.96	75.76	
A-CD	9.02	1	3.14	1.59	16.45	1.0000
B-solvent	7.37	1	3.14	−0.0573	14.80	1.0000
C-time	6.42	1	3.14	−1.01	13.85	1.0000
AB	1.24	1	4.44	−9.27	11.74	1.0000
AC	0.1700	1	4.44	−10.34	10.68	1.0000
BC	−7.15	1	4.44	−17.66	3.36	1.0000
A^2^	−18.62	1	4.33	−28.86	−8.38	1.01
B^2^	−1.67	1	4.33	−11.91	8.57	1.01
C^2^	−1.82	1	4.33	−12.06	8.43	1.01

**Table 5:** Model terms in RSM for lead.

Term	Standard error	VIF	*Ri* ^2^	Power (%)
A	0.3536	1	0.0000	68.1
B	0.3536	1	0.0000	68.1
C	0.3536	1	0.0000	68.1
AB	0.5000	1	0.0000	40.8
AC	0.5000	1	0.0000	40.8
BC	0.5000	1	0.0000	40.8
A^2^	0.4873	1.00588	0.0058	93.8
B^2^	0.4873	1.00588	0.0058	93.8
C^2^	0.4873	1.00588	0.0058	93.8

**Table 6:** Fit statistics.

Std. dev.	8.89
Mean	55.96
CV (%)	15.88
*R* ^2^	0.8507
Adjusted *R*^2^	0.6589
Predicted *R*^2^	−1.3436
Adeq. precision	5.9146

**Table 7:** RSM parameters for copper extraction.

Std	Run	Factor 1	Factor 2	Factor 3
		A: CD	B: solvent	C: time
		A/dm^2^	ml	Hrs
1	12	1	400	2
2	14	19	400	2
3	2	1	600	2
4	4	19	600	2
5	18	1	500	1
6	13	19	500	1
7	16	1	500	3
8	11	19	500	3
9	8	10	400	1
10	1	10	600	1
11	6	10	400	3
12	7	10	600	3
13	5	10	500	2
14	9	10	500	2
15	17	10	500	2
16	3	10	500	2
17	15	10	500	2

**Table 8:** Box–Behnken experimental design table for recovery of copper.

Std	Run	Factor 1	Factor 2	Factor 3
		A: CD	B: solvent	C: time
		A/dm^2^	ml	Hrs
1	12	1	400	2
2	14	19	400	2
3	2	1	600	2
4	4	19	600	2
5	18	1	500	1
6	13	19	500	1
7	16	1	500	3
8	11	19	500	3
9	8	10	400	1
10	1	10	600	1
11	6	10	400	3
12	7	10	600	3
13	5	10	500	2
14	9	10	500	2
15	17	10	500	2
16	3	10	500	2
17	15	10	500	2
18	10	10	500	2

**Table 9:** ANOVA quadratic model for copper.

Source	Sum of squares	DOF	Mean square	*F* value	*p* value	
*Model*	7763.50	9	862.61	155.08	<0.0001	Significant
A-CD	4608.00	1	4608.00	828.40	<0.0001	
B-solvent	544.50	1	544.50	97.89	<0.0001	
C-time	1922.00	1	1922.00	345.53	<0.0001	
AB	1.0000	1	1.0000	0.1798	0.6827	
AC	225.00	1	225.00	40.45	0.0002	
BC	25.00	1	25.00	4.49	0.0668	
A^2^	184.36	1	184.36	33.14	0.0004	
B^2^	279.27	1	279.27	50.21	0.0001	
C^2^	9.82	1	9.82	1.77	0.2206	
*Residual*	44.50	8	5.56			
Lack of fit	44.50	3	14.83			
Pure error	0.0000	5	0.0000			
*Total*	7808.00	17				

**Table 10:** Coefficients in terms of coded factors for copper.

Factor	Coefficient estimate	DOF	Standard error	95% CI low	95% CI high	VIF
Intercept	48.00	1	0.9629	45.78	50.22	
A-CD	24.00	1	0.8339	22.08	25.92	1.0000
B-solvent	8.25	1	0.8339	6.33	10.17	1.0000
C-time	15.50	1	0.8339	13.58	17.42	1.0000
AB	0.5000	1	1.18	−2.22	3.22	1.0000
AC	7.50	1	1.18	4.78	10.22	1.0000
BC	2.50	1	1.18	−0.2193	5.22	1.0000
A^2^	−6.50	1	1.13	−9.10	−3.90	1.02
B^2^	8.00	1	1.13	5.40	10.60	1.02
C^2^	1.50	1	1.13	−1.10	4.10	1.02

**Table 11:** Model terms in RSM for copper.

Term	Standard error	VIF	*R*i^2^	Power (%)
A	0.3536	1	0.0000	69.8
B	0.3536	1	0.0000	69.8
C	0.3536	1	0.0000	69.8
AB	0.5000	1	0.0000	42.1
AC	0.5000	1	0.0000	42.1
BC	0.5000	1	0.0000	42.1
A^2^	0.4787	1.01852	0.0182	95.4
B^2^	0.4787	1.01852	0.0182	95.4
C^2^	0.4787	1.01852	0.0182	95.4

**Table 12:** Fit statistics.

Std. dev.	2.36
Mean	49.33
CV (%)	4.78
*R* ^2^	0.9943
Adjusted *R*^2^	0.9879
Predicted *R*^2^	0.9088
Adeq. Precision	44.9395

## Data Availability

All the data used to support the findings of this study are included within the article.
